# Liquid Biopsy in Non-Small Cell Lung Cancer: Highlights and Challenges

**DOI:** 10.3390/cancers12010017

**Published:** 2019-12-19

**Authors:** Erika Rijavec, Simona Coco, Carlo Genova, Giovanni Rossi, Luca Longo, Francesco Grossi

**Affiliations:** 1 Medical Oncology Unit, Fondazione IRCCS Ca’ Granda Ospedale Maggiore Policlinico, Via Francesco Sforza, 28, 20122 Milan, Italy; ery80x@yahoo.it (E.R.); francesco.grossi@policlinico.mi.it (F.G.); 2 Lung Cancer Unit, IRCCS Ospedale Policlinico San Martino, Largo Rosanna Benzi 10, 16132 Genoa, Italy; giovanni.rossi.1689@gmail.com (G.R.); luca.longo@hsanmartino.it (L.L.); 3 Department of Medical, Surgical, and Experimental Sciences, University of Sassari, 07100 Sassari, Italy

**Keywords:** liquid biopsy, NSCLC, prognostic/predictive biomarkers, cfDNA/ctDNA, circulating miRNA, exosomes, tumor educated platelets

## Abstract

Non-small cell lung cancer is one leading cause of death worldwide, and patients would greatly benefit from an early diagnosis. Since targeted and immunotherapies have emerged as novel approaches for more tailored treatments, repeated assessments of the tumor biology have become pivotal to drive clinical decisions. Currently, tumor tissue biopsy is the gold standard to investigate potentially actionable biomarkers, but this procedure is invasive and may prove inadequate to represent the whole malignancy. In this regard, liquid biopsy represents a minimally invasive and more comprehensive option for early detection and investigation of this tumor. Today, cell-free DNA is the only approved circulating marker to select patients for a targeted therapy. Conversely, the other tumor-derived markers (i.e., circulating tumor cells, miRNAs, exosomes, and tumor educated platelets) are still at a pre-clinical phase, although they show promising results for their application in screening programs or as prognostic/predictive biomarkers. The main challenges for their clinical translation are the lack of reliable cutoffs and, especially for miRNAs, the great variability among the studies. Moreover, no established tool has been approved for circulating tumor cells and exosome isolation. Finally, large prospective clinical trials are mandatory to provide evidence of their clinical utility.

## 1. Introduction

In the last decade, tailored treatments for solid tumors, including non-small cell lung cancer (NSCLC), have emerged as novel therapeutic approaches, resulting in a clinically meaningful improvement in terms of survival. More specifically, oncogenic drivers such as activating mutations of the epidermal growth factor receptor (*EGFR*), as well as rearrangements of the anaplastic lymphoma kinase (*ALK*) or ROS proto-oncogene 1 (*ROS1*) are crucial targets for biological therapies in selected subgroups of NSCLC patients [1]. More recently, treatment with immune checkpoint inhibitors targeting the axis involving the programmed death protein 1 (PD-1) and its ligand (PD-L1) have revolutionized the management of a large proportion of NSCLC patients; with regards to immune checkpoint blockade, data on reliable predictive biomarkers are limited in comparison with targeted agents, although the expression of PD-L1 in tumor specimens is currently employed for the selection of patients considered eligible for first-line treatment with single-agent immune checkpoint inhibitors [2].

While the analysis of tumor tissue collected from biopsies is currently regarded as the current standard for the investigation of potentially actionable biomarkers, tumor biopsy is challenging as the procedure is invasive and may lead to the collection of inadequate samples, thus preventing proper analyses and requiring further attempts; furthermore, as NSCLC is characterized by non-negligible tumor heterogeneity, even an adequate biopsy may only be representative of a limited part of the whole malignancy. Finally, targeted antineoplastic therapies might lead to the onset of additional genetic alterations, eventually associated with acquired resistance to the ongoing treatments and potentially eligible for different targeted therapies. While such emerging alterations might be assessed through repeated biopsies, such procedures might not be feasible in the case of previously treated patients.

In this context, novel techniques designed to explore actionable targets might be a valuable addition to the available assessment methods. Liquid biopsy, which is based on the detection of tumor-related biomarkers from body-related fluids such as peripheral blood, represents an extremely appealing approach, due to its lack of invasiveness, easy accessibility, and good reproducibility. The aim of this review is to describe the current state of the art of liquid biopsies in NSCLC.

## 2. Circulating Tumor Cells

Circulating tumor cells (CTCs) are released by the primary solid tumor or its metastases into peripheral blood. They are rare, accounting for approximately one to 10 CTCs per 1 mL of whole blood [3]. Groups of more than two/three CTCs originate in clusters, which are also known as circulating micrometastases, circulating tumor microemboli, or circulating tumor aggregates. Individual CTCs have a longer life span (several hours) than CTC clusters, which can be blocked by small vessels [4].

In recent years, several efforts have been made to develop technical tools aimed at detecting CTCs, although there is no consensus on a universal assay. Currently, the CellSearch (Veridex LLC) assay is the only U.S. Food and Drug Administration (FDA) approved system for the detection and quantification of CTCs during the monitoring of patients affected by metastatic colorectal, breast, or prostate cancer. The CellSearch Circulating Tumor Cell Kit contains a ferrofluid-based capture reagent and immunofluorescent reagents. The ferrofluid reagent consists of nanoparticles with a magnetic core surrounded by a polymeric layer, coated with antibodies targeting anti-epithelial cell adhesion molecule (EpCAM) antigen for detecting CTCs. The identification and enumeration of CTCs is obtained by adding fluorescent reagents, which include anti-CK-phycoerythrin (PE), cytokeratin, and antiCD45 allophycocyanin (APC) specific for intracellular proteins, epithelial cells, and leukocytes, respectively, along with DAPI to stain the cell nucleus [5].

Isolation by size of epithelial tumor cells (ISET) is another method to gain CTCs that does not rely on the use of antibodies against tumor-associated markers. By this method CTCs are isolated by filtration, as a result of their different size and deformity compared to circulating blood leukocytes. Unlike the CellSearch, the filtration based technique allows for the collection of EpCAM negative cells, but it cannot detect CTCs with a very small size [6].

Farace et al. performed a comparison between CellSearch and ISET techniques for CTC detection in 60 patients with metastatic lung, breast, or prostate carcinoma, addressing some differences in the number of CTCs detected by the two assays. Indeed, by applying CellSearch and ISET assays, 18 (30%) and three (5%) patients, respectively, showed a negative result. The main limitations of CellSearch were observed in patients affected by NSCLC and prostate cancer. The lower CTC count achieved in these tumors can be explained by the epithelial–mesenchymal transition (EMT) of the cancer cells, which lose the expression of epithelial markers and gain the expression of cytoplasmic mesenchymal markers that are not detectable by CellSearch [7].

Recently Janning et al. performed a study to evaluate PD-L1 expression of CTCs of 127 samples from NSCLC patients, using a novel, label-independent microfluidic Parsortix TM system (ANGLE plc., Guildford, United Kingdom), which selects CTCs based on size and rigidity. The authors demonstrated that a dynamic increase in PD-L1 + CTCs could be associated with resistance towards immunotherapy [8].

In order to develop an assay able to detect a larger number of CTCs in NSCLC patients, Sharpenseel et al. evaluated the expression of different surface markers (i.e., EGFR and HER3). Several studies demonstrated that these proteins are upregulated in metastatic tissue and in CTCs compared to the primary tumor [9]. The authors concluded that the combination of EGFR/HER3 enrichment with the EpCAM-based CellSearch technique may detect a significantly higher number of CTCs in NSCLC patients by liquid biopsy [10].

Several studies investigated the prognostic role of CTCs in different types of tumors, including NSCLC [11]. The first of these studies was performed in 101 chemo-naïve advanced NSCLC patients and showed a prognostic significance of CTCs obtained with CellSearch. Blood samples were collected at baseline and after one cycle of standard chemotherapy. Progression-free survival (PFS) and overall survival (OS) were significantly longer in patients with less than five CTCs compared with patients with five or more CTCs (6.8 vs. 2.4 months, *p* < 0.001, and 8.1 vs. 4.3 months, *p* < 0.001, respectively). In multivariate analysis, CTC count was the strongest predictor of OS (*p* < 0.001) [12]. In line with previous studies, Yang et al. proved a relationship between CTCs and prognosis in 107 patients affected by advanced NSCLC harboring common activating *EGFR* mutations. Enrolled patients received first-line therapy with either erlotinib (150 mg) or gefitinib (250 mg). At baseline and on day 28 of treatment, serial blood samples were collected for detection of CTCs using CellSearch. The CTC measurements were classified into favorable (<5 CTCs) and unfavorable (≥5 CTCs) groups. Patients with the lowest count of CTCs at baseline and on day 28 showed a median PFS interval longer than patients in the unfavorable group (11.1 vs. 6.8 months, *p* = 0.009 and 11.6 vs. 6.3 months; *p* < 0.0001, respectively). At baseline the median time-to-treatment failure (TTF) was also significantly longer for patients in the favorable group compared with those in the unfavorable group (12.4 vs. 8.0 months, *p* = 0.0107). However, no significant difference in terms of TTF was observed between the two groups on day 28 (7.1 vs. 10.6 months; *p* = 0.061) [13]. On the contrary, Coco and colleagues showed an inverse but non significant relationship between baseline CTC number (6 CTC/3 mL of blood) and OS in 73 NSCLC patients (stage IIIB–IV) candidates for palliative first-line platinum-based chemotherapy. The authors hypothesized that a higher baseline number of heterogeneous CTC populations could exhibit different responsiveness to chemotherapy [14].

Recently, Chemi et al. evaluated the prognostic role of CellSearch-detected pulmonary venous (PV) CTCs, at the time of surgery, in 100 early-stage NSCLC patients, enrolled into the Tracking Non-Small Cell Lung Cancer Evolution through Therapy (TRACERx) trial. Although further studies are needed to confirm the clinical utility of PV-CTCs, the authors demonstrated that patients with detectable CTCs are more likely to experience disease recurrence [15].

The emergence of mutations conferring resistance to the use of tyrosine kinase inhibitors urged researchers to find methods that could detect and monitor genomic changes during EGFR targeted therapies. In 2008, Maheswaran et al. isolated CTCs from 27 patients with metastatic NSCLC, identifying the *EGFR* T790M mutation in 11/12 and 4/12 *EGFR* positive patients in CTCs and in matched plasma circulating cell-free DNA (cfDNA), respectively (*p* = 0.009). The authors concluded that the molecular analysis of CTCs could offer a valid option to monitor emerging mutations [16]. Very recently, a study by Zhang and colleagues showed a low detection rate for *EGFR* mutations (16.7%) at the single CTC level, albeit the sensitivity increased to 33.3% when the analysis was done on 10 CTCs per sample. Multiple CTCs are thus required to sensitively detect *EGFR* mutations [17].

In summary, the number of CTCs can be associated with the prognosis of NSCLC patients both in terms of PFS and OS. Moreover, mutation analysis on CTCs may contribute to the identification of resistance mechanisms. However, a reliable cut-off remains to be determined for a translation to the clinical practice. In addition, no technology for CTCs isolation and analysis has been approved by the FDA so far, so that a contribution of this approach to a more tailored therapy remains a challenge for the future.

## 3. Circulating Cell-Free DNA

Circulating cell-free DNA (cfDNA) derives from normal physiological tissue remodeling events, as for instance dividing cells such as gastrointestinal, epithelial, and hematological cells, and it can be detected at very low concentrations (5–10 ng/mL) in body fluids of healthy individuals [18]. cfDNA was determined to be approximately 180 bp in length, possibly associated with nucleosomes [19]. Noteworthy, there is strong evidence that supports the amount of cfDNA is significantly increased in patients affected by cancer compared to healthy individuals [20]. In recent decades, it has been widely documented that a fraction of cfDNA is, indeed, represented by the circulating tumor DNA (ctDNA), which mainly derives from necrosis and apoptosis of cancer cells, albeit active secretion has also been supposed [21]. The size of ctDNA fragments is shorter than that of cfDNA molecules that originate from non-malignant cells, although the causes of this difference in size have not been ruled out [22].

Current guidelines indicate plasma should be preferred to serum for the analysis of ctDNA [23]. Indeed, as a result of the leukocyte lysis during clotting, the contamination of germinal DNA is higher in serum than in plasma specimens [24]. In plasma specimens, the anticoagulant EDTA is suitable for cfDNA isolation, but the blood specimens must be processed within 6 hours from the sample withdrawal to prevent the release of normal DNA deriving from the lysis of blood cells. Moreover, the use of a leukocyte stabilization reagent allows even higher flexibility in the sample processing, which can be carried out within 2 days from the withdrawal without affecting ctDNA detection [25]. Once the blood sample is collected, plasma is usually isolated by at least two rounds of centrifugation at low speed and kept at −80 °C for long term storage [21]. ctDNA levels can vary greatly, ranging from 0.01% to more than 90% of total cfDNA [26]. Different high-sensitivity approaches including beads, emulsion, amplification, and magnetics (BEAMing), droplet digital PCR (ddPCR), next generation sequencing (NGS), and dedicated protocols are available to detect even a few copies of ctDNA [27,28,29].

Currently, a number of studies have shown that a high concentration of cfDNA at baseline represents a negative prognostic factor in terms of PFS and OS, irrespectively of patient characteristics like age and smoking habits, treatments and tumor histological type [13,30,31,32,33]. In this regard, Cargnin et al. performed a systematic review and meta-analysis to evaluate the impact of baseline cfDNA levels on the progression and survival of lung cancer patients. Interestingly, in the pooled analysis of OS, the authors reported an increased risk of death in those patients who had higher baseline cfDNA levels (*p* < 0.001), by evaluating a total of 1723 patients enrolled in 16 studies. Conversely, no association of baseline cfDNA with PFS (*p* = 0.29) has been reported in 640 patients enrolled in five studies [34].

As indicated by a growing body of evidence, one powerful employment of liquid biopsy is represented by its ability to detect genomic alterations in ctDNA, a tool that can be complementary to tissue molecular profiling. To date, several investigations have demonstrated *EGFR* gene mutations detected in cfDNA are highly concordant with those detected in tumor tissue of NSCLC patients, although with a variance (74–97%) mainly linked to the gene-test [35,36,37,38,39]. As a matter of fact, in 2016, ctDNA received U.S. FDA approval as the first liquid biopsy test for the identification of NSCLC patients with *EGFR* mutations, suitable for targeted therapy [40].

In the WJOG8114LTR multicenter, single-arm, prospective phase II study, the clinical significance of monitoring cfDNA was evaluated in 57 *EGFR* mutated, advanced lung adenocarcinoma patients during treatment with afatinib, an irreversible EGFR inhibitor. At baseline, 62.5% of patients were also positive for *EGFR* mutations in plasma and the rate of detection of *EGFR* mutations was higher among patients with systemic spread of the disease. Among positive patients, those with undetectable *EGFR* mutations in the cfDNA within four weeks after treatment showed a significantly longer PFS than those who did not achieve negative conversion (13.6 months versus 5.1 months, *p* < 0.0001) [41].

The usefulness of cfDNA mutation analysis has also been translated as a noninvasive approach to identify targetable mutations involved in the resistance to cancer therapy. One of the first studies that monitored the development of drug resistance by a serial analysis of cfDNA dates back to 2013. Here, the authors studied the cfDNA of six patients with metastatic cancer (three ovarian, two breast, and one lung cancer) both at baseline and during treatment by an innovative approach of whole exome sequencing, reporting an increase of mutant allele fraction in association with therapy-resistance [42]. In particular, in the lung cancer patient the cfDNA analysis showed the appearance of the T790M-*EGFR* mutation following gefitinib treatment, thus supporting the hypothesis of a selective pressure linked to the treatment. Starting from these encouraging data, several efforts have been made in this field also in NSCLC. For example, Thress et al. identified a novel *EGFR* mutation (C797S) as a specific acquired resistance mechanism to AZD9291 by screening the cfDNA from 15 *EGFR* T790M positive patients during treatment with this EGFR inhibitor [43,44].

Besides *EGFR* mutational status, both *ALK* rearrangements and point mutations linked to resistance to tyrosine kinase inhibitors were successfully evaluated in plasmatic cfDNA of lung cancer patients [45,46,47,48]. In this context, Bordi et al. performed a screening of the cfDNA from 20 *ALK* positive NSCLC patients treated with crizotinib who suffered progression. Mutational analysis of cfDNA at the progression time identified novel *ALK* point mutations in five out of 20 patients. Notably, the authors also observed a positive correlation between the decrease of *ALK* acquired mutations in the cfDNA and the radiological response, suggesting that monitoring of resistance *ALK* mutations can be useful as a response parameter [45]. Similarly, another study demonstrated the efficacy of cfDNA analysis to detect *ALK* fusions and identify resistance mechanisms by a 70-gene NGS panel (Guardant360 test) [46]. More recently, a comprehensive cfDNA analysis with the Guardant360 on 282 prospectively enrolled patients with previously untreated metastatic NSCLC showed that the genotyping of cfDNA for eight recommended markers, including *EGFR*, *BRAF*, and *ERBB2* mutations, *ALK*, *ROS1*, and *RET* fusions, and *MET* aberrations (amplification and exon 14 skipping variants) was a clinically relevant alternative to tissue genotyping. In particular, 60 (21.3%) and 77 (27.3%) patients tested positive for tissue and cfDNA genotyping, respectively. Among the 60 patients with a tissue positivity for at least one of the eight markers, 48 (80%) were concordant with the cfDNA results, and this concordance reached a percentage greater than 98% if considering the FDA-approved targets (i.e., *EGFR*, *ALK*, *ROS1*, *BRAF*) only [49].

Overall, cfDNA is generally found at higher levels in subjects with cancer than in healthy individuals. Although cfDNA concentration has been proposed as a predictor of survival in NSCLC, no universal cutoff is currently available, avoiding its translation to the clinic. The most promising results concern the cfDNA mutational analysis. Indeed, it has emerged that cfDNA may be exploited to identify specific mutations and molecular targets for personalized therapies. However, the concordance between the mutational status of cfDNA and tumor DNA is still not optimal. Aside from the tumor heterogeneity, the low portion of ctDNA in the plasma may also be involved in this issue. In this regard, other body fluids have been evaluated as an alternative source of tumor derived DNA. In fact, cfDNA from fluids in sites adjacent to metastases such as cerebrospinal fluid, pleural effusion, and ascites was found at higher levels, resulting in a more effective method in for detecting relevant mutations [50].

## 4. Circulating miRNAs

Besides cfDNA, circulating RNA molecules have also been described in different biological fluids. In particular, a major interest has focused on microRNAs (miRNA) [51,52]. MiRNAs are short non-coding single stranded RNA molecules (19 to 22 nucleotides in length) that target complementary mRNA sequences mainly at the 3′-untranslated region, thus regulating gene expression at the post transcriptional level [53]. Each miRNA can regulate several genes and their dysregulation has been linked to cancer. Indeed, miRNAs can function as both tumor suppressors and oncogenes, and the impairment of their expression might ultimately lead, for instance, to tumor formation, cell proliferation, invasion, cell death, and angiogenesis [54]. Despite several studies demonstrating the existence of cell-free miRNAs in body fluids, the exact mechanisms of release of miRNAs into the extracellular environment are not yet completely understood. Emerging evidence has demonstrated that the release of miRNAs into the extracellular space is not an exclusively “passive” mechanism, linked to cell death and lysis (e.g., necrosis and apoptosis), but also an “active” one, which is involved in intercellular communication [55].

In the latter case, miRNAs are packed into extracellular vesicles such as exosomes and microvesicles or, alternatively, coupled with Argonaute2 (Ago2) protein or high-density lipoprotein (HDL) and released to the extracellular environment [56,57]. Several studies have shown circulating miRNAs may reflect the biology of the tumor, and their expression may relate to tumor development, progression, and metastases. Circulating miRNAs are thus candidate biomarkers in screening programs as potential predictors of diagnosis, prognosis, and response to therapies [58]. It is noteworthy that circulating miRNAs can discriminate lung cancer patients from healthy individuals with excellent performances [59,60,61,62,63,64,65]. In this context, two large retrospective studies reported two miRNA signatures that may be used as potential noninvasive biomarkers for early detection of lung cancer, showing a significant reduction of the false-positive rate of low-dose computed tomography (LDCT) [62,63]. In the first study, Sozzi et al. retrospectively profiled the expression of 24 circulating miRNAs in plasma samples from 997 individuals enrolled in a lung cancer screening clinical trial. The microRNA signature classifier showed an optimal performance with a five-fold decrease of LDCT false-positive rate [62]. Similarly, Montani and colleagues tested a miRNA signature (miRtest) based on the expression of 15-miRNAs in serum samples from 1115 individuals previously enrolled in a lung cancer screening program [63]. The overall accuracy, sensitivity, and specificity of both miRNA signatures were good and quite similar across the studies. In addition, despite two miRNA signatures being obtained from different specimens (plasma and serum), five miRNAs were shared by both signatures. Recently, a meta-analysis, including almost 14,000 individuals (6919 patients and 7064 controls) from 134 studies has shown that the circulating miRNAs had an optimal diagnostic accuracy (area under the curve AUC: 0.90) with high sensitivity (82%) and specificity (81%). The pooled analysis identified a 4-miRNA panel (miR-21-5p, miR-223-3p, miR-155-5p, and miR-126-3p) as a potential biomarker for lung cancer screening [66]. The authors also reported serum samples as a more suitable source of tumor derived miRNAs than plasma samples. 

Besides their application as a screening tool, the levels of some serum miRNAs can be a reliable predictor of survival in either early stage patients before the surgical resection or patients with metastatic disease [67,68,69,70]. In 2010, by using a sequencing approach, Hu and colleagues screened the serum miRNome of 60 early NSCLC patients (30 long survivors with a mean survival time of 49.5 months, and 30 short survivors with a mean survival time of 9.5 months), identifying 11 miRNAs that were deregulated more than five-fold between the two survival groups. Then, the validation of these miRNAs in an independent cohort of 243 early stage patients by single qPCR assays confirmed that the levels of expression of four out of 11 miRNAs (miR-486, miR-30d, miR-1, and miR-499) were significantly associated with the OS [65]. In 2018, Zhang et al. built two distinct miRNA signatures by profiling the serum miRNAs of more than 150 early stage patients. These signatures were based on an miRNA expression ratio, which could improve the prediction of disease recurrence and survival rate compared with clinical factors. The further validation in 246 tumor samples showed miRNA-150 level was significantly associated with the 5-year survival (HR, 0.87; 95% CI, 0.76–0.99; *p* = 0.04) [67]. In addition, by using both in vitro and in vivo models, the authors also demonstrated that the miRNA-150 could enhance cell proliferation and migration by silencing *SRCIN1*, a tumor suppressor gene. Moreover, the same authors had previously analyzed the expression profile of serum miRNAs in a large cohort of 391 advanced stage (III and IV stage) patients, identifying a 17-miRNA risk score. Notably, patients with a high risk miRNA profile in serum showed a 2.5-fold increased risk of death compared with those with a low risk score (95% CI: 1.8–3.4; *p* = 1.1 × 10^−7^) [66]. Very recently, a similar study including more than 500 individuals with metastatic lung cancer, identified five serum miRNAs (miR-191, miR-28-3p, miR-145, miR-328, and miR-18a) significantly associated with the 3-year overall survival [68].

Although all previous findings, and others as well, are quite encouraging, representing a novel and less invasive option for the early detection, diagnosis, and prognosis of NSCLC, circulating miRNA signatures exhibit a high variability, which seems to depend primarily on the cohort as well as on the technologies employed. Still, there are many constraints that limit their clinical utility. For instance, pre-analytical phases of quantification and detection of miRNAs need to be validated to render reliable results [71]. Moreover, among the most critical concerns, circulating miRNAs do not show specificity for a type of cancer, and a big issue is still represented by the lack of a valid method for the normalization of results. Nonetheless, should these and other issues be resolved, circulating miRNAs may account for a promising cancer biomarker in the near future.

## 5. Exosomes

Exosomes are the smallest extracellular vesicles, with a size of 40–100 nm. They originate from the endosomal system, carrying DNA, mRNAs, noncoding miRNAs, proteins, and lipids [72]. Worthy of note, RNA molecules entrapped within exosomes are more resistant than the free form to the activity of RNases [73]. All cell types, including tumor cells, seem to be able to release exosomes into the extracellular space [74]. Once released, exosomes can act as messengers, targeting cells by merging with the plasma membrane or by interacting with protein receptors [75]. Exosomes can ideally be isolated from almost all body fluids, but due to their nano-size, the purification of high yield extracellular exosomes is challenging. Currently, a number of techniques have been employed for exosome isolation, including physical methods (e.g., ultracentrifugation, density gradient separation, ultrafiltration, size exclusion chromatography), chemical precipitation methods (e.g., polymeric-based precipitation), and biological assays (e.g., immune-bead isolation) [70]. Nevertheless, each of the aforementioned technologies has advantages and disadvantages. The selection of the best exosome-isolation tool depends primarily on the type of molecules that should be screened as well as on the lab-equipment, such as the availability of ultracentrifuges. For instance, since HDL–miRNA complexes are co-purified with exosomes whilst using a density separation tool, this methodology should be avoided in studies that focus on exosomal miRNAs only. In addition, to improve the reproducibility across laboratories, commercial kits are now available for the isolation of exosomal RNA and proteins [70,76]. Evidence supports exosome number being higher in patients with cancer than in healthy controls [77]. In a recent study, by screening the pulmonary vein-derived exosomes in a cohort of 72 resected NSCLC patients, Navarro and colleagues showed that exosome size (cut-off: 112 nm) was able to discriminate relapsed patients with a higher accuracy than clinical data (stage and lymph node involvement). In the multivariate analysis, exosome size resulted an independent risk factor for both time to relapse (HR: 6.66; 95% CI, 2.06–21.51; *p* = 0.001) and OS (HR: 4.55; 95% CI, 1.43–14.49; *p* = 0.010) [78]. Whatever their dimension, exosomes have an active role in the process of carcinogenesis, progression, and metastasis of several tumors, including NSCLC, which is exerted by transferring their cargo. For instance, exosomes can promote angiogenesis by transferring proteins such as fibroblast growth factor (FGF), vascular endothelial growth factor (VEGF), IL-6, and IL-8, and by stimulating vascular endothelial cells via miRNAs [79,80]. Yongjian and colleagues demonstrated that miR-660-5p, a miRNA known for its role in the progression of breast cancer, is also expressed at high levels in NSCLC patients both in exosomes and in blood plasma, compared with healthy controls. In vitro and in vivo experiments showed miR-660-5p promotes NSCLC progression by targeting KLF9, which is a gene of the KLF (Krüppel-like factors) family that regulates several physiological processes, including differentiation, proliferation, and apoptosis [81]. Similar to the circulating miRNAs, exosomal miRNAs were also shown as promising and effective noninvasive candidate biomarkers for early NSCLC diagnosis. Li et al. demonstrated that the expression of noncoding RNA growth arrest-specific transcript 5 (GAS5) in circulating exosomes (Exo-GAS5) could be an ideal non-invasive blood-based tumor marker for the identification of patients with early-stage NSCLC. The expression of Exo-GAS5 was significantly lower in NSCLC patients than in healthy controls (*p* < 0.001) and higher in early-stage compared with advanced-stage NSCLC patients (*p* = 0.045) [82]. Recently, Zhang et al. demonstrated that the expression of exosomal miR-17-5p was significantly higher in NSCLC patients than in healthy controls (*p* < 0.001). In this study, the authors built a four-molecule panel that included miR-17-5p in combination with three circulating tumor markers, which are the carcinoembryonic antigen (CEA), cytokeratin 19 fragment (CYFRA21-1), and squamous cell carcinoma antigen (SCCA). The diagnostic performance of this panel was verified, and the ROC analysis demonstrated an AUC of 0.860 (95% CI = 0.802 to 0.906, sensitivity = 63%, and specificity = 93%) and 0.844 (95% CI = 0.766 to 0.904, sensitivity = 76%, and specificity = 77%) in the training and validation sets, respectively [83].

The usefulness of exosomal RNA has also been demonstrated in the identification of somatic mutations of tumor origin. Krug et al. screened exosomal RNA and cfDNA in parallel from a cohort of 84 *EGFR* positive NSCLC patients (stage IIIB, IV) enrolled in TIGER-X (NCT01526928), a phase 1–2 trial to receive rociletinib [84]. The authors showed that exosomal RNA was significantly more sensitive to detect activating *EGFR* mutations (98%) and *EGFR*-T790M (90%) compared to the matched cfDNA (82% for activating EGFR mutations and 84% for *EGFR*-T790M). Interestingly, the most promising findings were in a subgroup of patients with intrathoracic metastasis (21/84), where the exosomal RNA allowed the identification of 14 and five positive cases out of 21 patients for *EGFR* activating mutations and T790M, respectively, compared to five and three positive samples detected in the paired cfDNA. Exosomes can also be a source of DNA. In particular, in the exosomal DNA derived from human melanoma (*BRAF* positive) and NSCLC (*EGFR* positive) cell lines, Thakur and colleagues were able to detect the very same driver mutations of the parental cell line [85]. Finally, recent evidence supports EGFR protein can be transferred from human carcinoma cells to endothelial cells through exosomes, thus leading to the activation of the downstream pathway as well as the VEGF pathway [86].

In conclusion, all previous studies indicate exosomes are ideally one of the most promising liquid biopsy markers in the cancer field. Indeed, exosomes are higher in cancer patients and their size might, for instance, help the clinician in selecting patients with unfavorable outcomes. Exosomes can be seen as shuttles carrying tumor-derived molecules (RNA and DNA), and they are emerging as promising biomarkers both in lung cancer diagnosis and prognosis. In particular, the analysis of exosomal nucleic acids showed them to be more sensitive to identifying relevant mutations than that of cfDNA. However, some potential drawbacks linked to the isolation procedure are lessening their transfer to a clinical context. In addition, as already reported on circulating miRNAs, despite several exosomal miRNA signatures having been proposed for lung cancer screening, no or minimal overlap occurs across the studies, strongly limiting their clinical usefulness.

## 6. Tumor Educated Platelets

Platelets are small enucleated cell fragments derived from megakaryocytes. They represent the second most common type of blood cell (150–400 × 10^9^ per liter of whole blood) and their average lifespan is quite short (8–10 days). Several structural elements are typical of the platelets, including the open canalicular system derived from invaginations of the surface membrane and the dense tubular system, a remnant of the rough endoplasmic reticulum. The cytoplasm contains several organelles including ɑ-granules, dense-granules, lysosomes, and mitochondria [87]. Platelets are achieved from whole blood and collected by a two-step centrifugation protocol at room temperature, making their isolation a very simple procedure [88]. Platelets play a crucial role in the coagulation process, although they are also known to regulate tumor angiogenesis [89]. Tumor cells produce several growth factors, such as granulocyte–macrophage colony-stimulating factor, granulocyte colony-stimulating factor, and cytokines as for instance, interleukin-1 and interleukin-6 able to promote thrombocytosis [90]. The strong interaction among platelets, tumor, and its microenvironment give rise to tumor educated platelets (TEP). Platelets are in fact able to internalize bio-molecules released by tumor cells such as proteins, mRNA, and miRNAs. Platelet derived growth factor, VEGF, and other growth factors produced by tumor cells are known to change the expression of mRNA present in platelets, leading to a specific spliced mRNA signature [91,92].

Sheng and colleagues analyzed RNA-sequencing data of TEPs in 402 NSCLC patients and 231 healthy controls. The authors identified 48 genes having a key role in tumorigenesis and cancer progression that can accurately predict NSCLC, suggesting that TEPs might be useful for the early detection of NSCLC [93]. A recent study showed that particle-swarm optimization-enhanced algorithms enable efficient selection of gene panels from platelet RNA-sequencing libraries, obtaining an accurate TEP-based detection of early stage or advanced NSCLC regardless of smoking history, age, inflammatory conditions, and whole-blood storage time [94]. To conclude, although TEPs are promising as a liquid biopsy source, large prospective studies and clinical trials will be necessary in the future to give evidence of their clinical value. 

## 7. Conclusions

The applications of liquid biopsy for the diagnosis and treatment decision of NSCLC patients are increasing. Compared to invasive tissue biopsy, the minimal invasiveness and the low cost of liquid biopsy allow repeatable evaluations of cancer patients, making it a very useful tool in the clinical practice. Evidence indicate liquid biopsy can be applied to dynamically evaluate resistance mutations during treatment with EGFR and ALK inhibitors. To date, the screening of cfDNA/ctDNA is the only valuable circulating marker for the selection of patients eligible for treatment with a TKI, when the tumor biopsy is not feasible. In addition to the cfDNA, other circulating markers such as CTCs, miRNAs, exosomes, and TEPs appear to be reliable surrogates to understand the tumor biology. As a consequence, they are likely to eventually be translated to the clinic in the near future. Indeed, all the encouraging findings reviewed herein paved the way to activate several clinical trials aimed at evaluating circulating tumor derived biomarkers, alone or in combination, in different screening programs (Table 1), and at monitoring drug resistance (Table 2). 

Recently, the introduction of immune checkpoint inhibitors (ICI) against PD-1 and PD-L1 has radically modified lung cancer care. However, except for PD-L1 tissue expression (>50%) that is used to select advanced NSCLC patients to receive pembrolizumab in the first line setting, no reliable prognostic or predictive marker is presently acknowledged for other ICIs. In this context, in preclinical studies, we found that circulating markers may have significant results [29,95,96]. Moreover, the correlation between PD-L1 expression on CTCs and prognosis of advanced patient cohorts treated with immunotherapy has been explored. Notably, PD-L1 positive CTCs are associated with a bad prognosis [97,98].

In addition, the evaluation of tumor mutational burden (TMB), which is the number of somatic mutations in the genome coding regions (Mut/Mb), has been explored and is currently been tested in the cfDNA, to identify patients who can benefit from immunotherapy [99,100,101,102]. However, the first results on the concordance between tissue and cfDNA are contrasting [103]. One of the major issues is the lack of standardization in the TMB evaluation. For instance, the sequencing panels covered different genomic regions. In addition, the TMB calculation, intended as the type of somatic mutations (e.g., passenger or driver mutations), varied among the studies. Last but not least different cut-offs were used to stratify patients. 

In conclusion, although inherent marker-dependent drawbacks are inevitably encountered, each marker has provided evidence for a prospective use in the early detection of lung cancer (Table 3). Hence, liquid biopsy may potentially play a helpful role in the future to guide clinicians in the management of this fatal disease. However, large prospective multi-institutional clinical trials are needed to provide the evidence that liquid biopsy can be a valid alternative to tumor tissue biopsy. In the forthcoming years, liquid biopsy could routinely be performed to diagnose cancer at an early stage by a blood-test, before any symptomatology or radiological assessment, when the chances for a cure are higher.

## Figures and Tables

**Table 1:** Active studies that explore the feasibility and utility of liquid biopsy in early stage NSCLC.

NCT	pts	Study Type	Title/Scopus	Study Completion	Markers
NCT03721120	286	Ran	Feasibility and Clinical Relevance of Liquid Biopsy in Patients with Suspicious Metastatic Lung Cancer (LIBELULE)	July 2021	ctDNA
NCT03553550	500	ObP	Role of Circulating Tumor DNA (ctDNA) From Liquid Biopsy in Early Stage NSCLC Resected Lung Tumor Investigation (LIBERTI)	June 2024	ctDNA
NCT02906852	50	int	Evaluation of NSCLC Genetic Heterogeneity in Patients with Operable Early Stage Disease and Prediction of Clinical Relapse Using a Personalized “Liquid Biopsy”	December 2021	ctDNA, CTCs
NCT03479099	111	ObP	Clinical Utility of Combined CTC and ctDNA Assay in the Diagnosis of Primary Lung Cancer	March 2019	CTCs, cfDNA
NCT02511288	900	ObP	Liquid Biopsies in Patients Presenting Non-Small Cell Lung Cancer (LIBIL)	December 2026	cfDNA, CTCs, circulating miRNA
NCT03838588	200	ObP	Tracking Genomic Cancer Evolution in Patients for Stage IB, II, and IIIA Non-Small Cell Lung Cancer After Radical Resection: The Tracking Molecular Evolution for NSCLC (T-MENC) Study	December 2021	ctDNA
NCT03774758	590	ObP	Circulating Tumor DNA for Risk Stratification in Lung Cancer Screening	December 2023	ctDNA
NCT03576937	210	ObP	Achieving Value in Cancer Diagnostics: Blood Versus Tissue Molecular Profiling: A Prospective Canadian Study (VALUE)	September 2020	ctDNA

Abbreviations: Ran: Randomized; Int: Interventional; ObP: Observational prospective; CTC: circulating tumor cells; ctDNA: circulating tumor DNA; NSCLC non-small cell lung cancer.

**Table 2:** Active studies that explore the feasibility and utility of liquid biopsy in advanced NSCLC harboring driver mutations.

NCT	pts	Study Type	Title/Scopus	Study Completion	Markers
NCT02778854	200	ObP	Liquid Biopsy for Detection of Driver Mutation in NSCLC November 2020 ddPCR	November 2020	ctDNA
NCT02771314	48	int	A Longitudinal Study Evaluating Molecular Changes Associated with Resistance to First and Third (AZD9291) Generation EGFR TKIs in Patients with EGFR Mutant NSCLC Using “Liquid Biopsy”	December 2020	CTC and ctDNA
NCT03865511	66	Int	Phase 2 Study Evaluating Mechanisms of Resistance on Tumor Tissue and Liquid Biopsy in Patients with EGFR Mutated Nonpretreated Advanced Lung Cancer Receiving OSimErtinib Until and Beyond Radiological Progression: the MELROSE Trial	July 2024	ctDNA
NCT03833934	300	ObP	ALCMI-011: Study of Plasma Next Generation Sequencing for Assessment, Characterization, Evaluation of Patients with ALK Resistance (SPACEWALK)	February 2021	ctDNA

Abbreviations: Ran: Randomized; Int: Interventional; ObP: Observational prospective; CTC: circulating tumor cells; ctDNA: circulating tumor DNA; NSCLC non-small cell lung cancer.

**Table 3:** Summary of the main highlights and challenges of the circulating markers.

	Source	Highlights	Challenges
**CTCs**	Peripheral blood	CTC number correlates with PFS and OS; Dynamic monitoring of molecular alterations during therapy; Assessment of tumor markers (e.g., PD-L1) at baseline and during therapy;	No FDA-approved technology for isolation; Rare events; Lack of reliable cut-off;
**cfDNA ctDNA**	Plasma, Serum, Cerebrospinal fluid, Pleural effusion, Ascites	Flexibility in processing (stable for up to 2 days in blood sample); No specific device required for isolation (benchtop centrifugation); cfDNA level correlates with PFS and OS; ctDNA reflects tumor heterogeneity; Dynamic monitoring of molecular alterations during therapy; Identification of novel targetable resistance mutations;	Lack of reliable cut-off; Contamination of germinal cfDNA; cfDNA level reflects changes in ctDNA and patient characteristics as well as medical conditions; Low portion of ctDNA in plasma; Not all gene mutations are expressed in ctDNA;
**Ci-miRNAs**	Plasma, Serum	More resistant to RNases than mRNA; No specific device required for isolation (benchtop centrifugation); Ci-miRNAs reflect the biology of the tumor; Ci-miRNAs can discriminate healthy individual from patients and early stage from advanced patients; Ci-miRNA expression correlates with tumor development, progression and metastases;	Lack of standardization (e.g., RNA isolation, quantification); High variability; Lack of large prospective studies;
**Exosomes**	Almost all body fluids	Stable sources of tumor-derived genetic material (e.g., DNA, RNA, miRNAs, and proteins); Exosome number is higher in patients than in healthy controls; Exosome size correlates with unfavorable outcome; Exo-miRNAs can discriminate healthy individual from patients and early stage from advanced patients; Exo-RNA and Exo-DNA have a higher sensitivity in detection of somatic mutations than plasma ctDNA;	Unreliable isolation procedures; Minimal overlap among exo-miRNA signatures; Lack of large prospective studies;
**TEP**	Peripheral blood	No specific device required for isolation (benchtop centrifugation); TEP-RNA reflects tumor transcriptome; TEP-RNA can discriminate healthy individual from patients and early stage from advanced patients;	Lack of large retrospective and prospective studies;

Abbreviations: CTC: circulating tumor cells; PFS: progression free survival; OS: overall survival; cfDNA: circulating cell-free DNA; ctDNA: circulating tumor DNA; ci-miRNA: circulating miRNA; Exo-DNA: Exosome DNA; Exo-RNA: Exosome RNA; TEP: tumor educated platelets.
